# A Fatal Case of Dengue-Associated Hemophagocytic Lymphohistiocytosis and Retroperitoneal Hematoma in a Patient With Autoimmune Hemolytic Anemia

**DOI:** 10.7759/cureus.15001

**Published:** 2021-05-13

**Authors:** Chee Yik Chang

**Affiliations:** 1 General Medicine, Hospital Sultanah Aminah, Johor Bahru, MYS

**Keywords:** dengue fever, hemophagocytic lymphohistiocytosis, autoimmune hemolytic anemia, retroperitoneal hematoma, dengue shock syndrome

## Abstract

Hemophagocytic lymphohistiocytosis (HLH) is a rare but potentially life-threatening complication of dengue infection which necessitates early diagnosis and treatment to improve patient outcomes. Severe dengue infection complicated by HLH may require interventions such as systemic corticosteroids, intravenous immunoglobulin, or chemotherapy. Here, we report a case of concurrent dengue-induced HLH and flare of underlying autoimmune hemolytic anemia (AIHA). The disease was refractory to treatment including corticosteroids and intravenous immunoglobulin. The course of illness was later complicated by dengue shock syndrome, severe liver dysfunction, and a large retroperitoneal hematoma. Unfortunately, the patient succumbed on day 10 of illness.

## Introduction

Dengue virus is a single-stranded positive-sense ribonucleic acid virus and belongs to the family *Flaviviridae*, genus Flavivirus. The dengue virus has four distinct serotypes, namely dengue virus type 1 (DEN-1), DEN-2, DEN-3, and DEN-4 [1]. In recent years, there have been increasing cases of hemophagocytic lymphohistiocytosis (HLH) associated with dengue infection, leading to high mortality rates [2]. HLH is a hyperinflammatory condition that is characterized by macrophage activation with phagocytosis of blood cells in the bone marrow and subsequent cytokine storm, leading to organ dysfunction and death [2,3].

HLH rarely occurs in the presence of autoimmune hemolytic anemia (AIHA). Wang et al. reported an unusual case of pregnancy-induced HLH with concomitant AIHA that resolved spontaneously after the termination of pregnancy [4]. Thus far, there has been no reported case on AIHA and HLH following dengue infection. Here, we report a case of severe dengue complicated by acute hemolysis with underlying warm AIHA, HLH, and retroperitoneal hematoma.

## Case presentation

A 59-year-old lady with non-insulin-dependent diabetes mellitus presented to Hospital Sultanah Aminah, Johor, Malaysia with fever, myalgia, and headache of two days duration. She reported no bleeding tendency or rash. She had a known history of warm AIHA diagnosed two years prior. Her condition was deemed to be in remission during the last hematology clinic follow-up. She was on oral prednisolone 5 mg daily and azathioprine 50 mg daily.

Upon arrival to the hospital, she appeared alert and pale. Her blood pressure was 103/55 mmHg and her heart rate was 90 beats per minute. She was febrile, not tachypnoeic, and the oxygen saturation was 98% while breathing ambient air. Her initial full blood count showed a hemoglobin of 7.2 g/dL, while the white cell and platelet counts were within the normal limits. Due to the clinical suspicion of dengue infection, a rapid dengue non-structural (NS)-1 antigen test (SD Bioline Dengue Duo kit, Standard Diagnostics Inc., Seoul, Korea) was requested and shown to be positive, while dengue immunoglobulin M (IgM) and IgG were negative. Dengue virus type 3 (DEN-3) was later identified by reverse transcription-polymerase chain reaction (RT-PCR) assay. Peripheral blood film examination revealed active hemolysis with elevated reticulocyte count. The liver transaminases were elevated (alanine aminotransferase (ALT) 70 U/L, aspartate aminotransferase (AST) 137 U/L) while the renal function test was normal. The serum ferritin and lactate dehydrogenase (LDH) on admission were 16851 mcg/L and 655 U/L, respectively.

In the ward, the patient received maintenance intravenous fluid therapy (1-1.5cc/kg/hour) and she was started on intravenous hydrocortisone 100 mg every eight hours in view of active AIHA. Since admission, she was noted to be febrile until attaining defervescence on day five of illness. She remained normotensive and did not exhibit any signs of plasma leakage or hyperinflammation. Subsequently, the hematological and biochemical indices suggested reducing hemolysis.

However, her fever recurred on day 6-7 of illness with a high temperature recorded at 38.9°C. Serial blood cultures were negative and the C-reactive protein level was not raised. At this point in time, the liver function tests were markedly deranged (ALT 281 U/L, AST 1426 U/L, LDH 3793 U/L) and the serum ferritin level was >100000 mcg/L. Besides, hypofibrinogenemia and high triglyceride levels were also observed (Table 1). There was no organomegaly noted on physical examination. As a result, a clinical diagnosis of HLH was made as the calculated HScore was 197, with an 84% probability of HLH [5]. Intravenous hydrocortisone was then changed to intravenous methylprednisolone 500 mg daily, and intravenous N-acetylcysteine was also started.

**Table 1:** Serial blood investigations of the patient ALT: alanine aminotransferase; AST: aspartate aminotransferase; LDH: lactate dehydrogenase.

	Day of illness								
	D2	D3	D4	D5	D6	D7	D8	D9	D10
Haemoglobin (g/L) (12-15)	7.2	7.6	7.4	7.0	8.1	8.5	9.9	7.0	6.3
Haematocrit (36-46)	17	19	18	17	22	23	22	20	19
White cell (x10^9^/L) (4-10)	4.6	3.9	5.8	4.7	3.6	3.8	3.6	6.3	10
Platelet (x10^9^/L) (150-410)	178	166	127	127	105	94	51	49	74
ALT (U/L) (5-33)	70	73	58	60	70	132	310	353	1006
AST (U/L) (5-32)	137	148	187	233	267	525	1426	1609	4370
LDH (U/L) (135-214)	655	837	1186	1555	1911	2054	3793	4954	5166
Total bilirubin (μmol/L) (<21)	57	53	39	30	24	21	47	103	134
Direct bilirubin (μmol/L)	25		18		11				
Indirect bilirubin (μmol/L)	32		21		13				
Ferritin (μg/L) (13-150)	16851						>100000	>100000	>100000
Fibrinogen (g/L) (2.2-3.9)							1.3	1.4	0.8
Triglyceride (mmol/L) (1.7-2.3)							2.7	1.6	2.0
Creatinine (μmol/L) (44-80)	45	49	41	36	39	33	30	49	96

**Figure 1:**
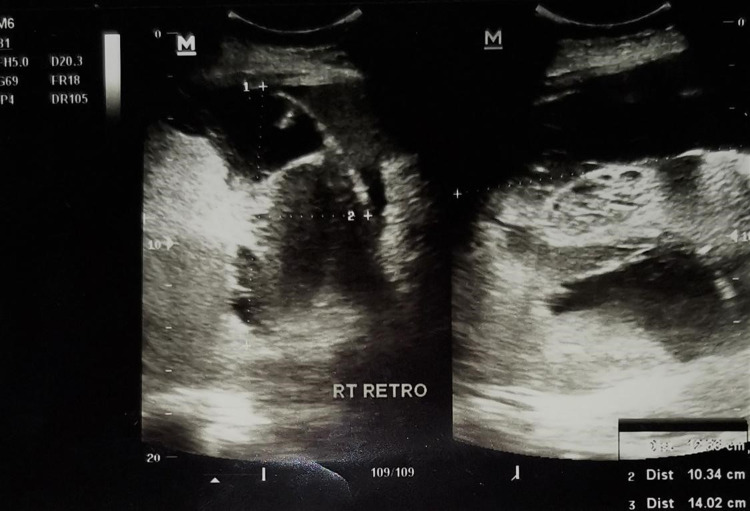
Abdominal ultrasonography showed a right retroperitoneal hematoma

However, the patient did not improve despite three days of methylprednisolone with persistent fever, serial serum ferritin levels persistently >100000 mcg/L, and severe liver dysfunction. Therefore, intravenous immunoglobulin 4 mg/kg was given on day 8 of illness. Bone marrow examination was not performed due to her unstable condition. On day 9 of illness, she developed hypotensive shock that required multiple fluid boluses and inotropic support. She was subsequently intubated due to respiratory distress. She was noted to have increasing abdominal distension and a concurrent drop in hemoglobin of 3 g/dL from the previous day (9.9 to 7.0 g/dL). Urgent abdominal ultrasonography revealed a large retroperitoneal hematoma measuring 10 x 10 x 14 cm (Figure 1). She was transfused with a total of eight units of packed cells and two cycles of disseminated intravascular coagulation (DIC) regimes. Her condition was deemed critically ill and unfit for surgical management of retroperitoneal hematoma. She finally succumbed on day 10 of illness.

## Discussion

Dengue virus belongs to the genus flavivirus within the *Flaviviridae* family and is the cause of dengue infection. There are an estimated 50 million dengue infections and 500,000 individuals are hospitalized with dengue hemorrhagic fever each year, predominantly in Southeast Asia, the Pacific, and the Americas [1,6]. The mortality rate associated with dengue infection was reported to be around 20,000 deaths per year [6]. Severe dengue is the major cause of death resulting from plasma leakage, fluid accumulation causing respiratory distress, severe hemorrhage, or organ failure. Furthermore, severe dengue can be complicated by secondary HLH [2,7].

Infection with the dengue virus has been increasingly recognized as an important cause of secondary HLH, and the mortality rate was as high as 43% [2,7]. HLH is a hyperinflammatory condition that is characterized by macrophage activation with phagocytosis of blood cells in the bone marrow, and cytokine storm leading to organ dysfunction and death. To date, the exact mechanisms by which viruses are implicated in the pathogenesis of HLH remain unproven. Viruses may promote the development of HLH by evading immune recognition, inhibiting apoptotic pathways, and interfering with cytokine regulation [3]. HLH can be classified into primary (genetic) or secondary (acquired). The latter is caused by either a viral infection, autoimmune disease, or neoplastic condition [2]. Diagnosis of HLH requires fulfillment of at least five of the eight criteria as listed: fever, splenomegaly, cytopenia affecting at least two of three lineages in peripheral blood, ferritin ≥500 μg/L, hypertriglyceridemia and/or hypofibrinogenemia, hemophagocytosis in bone marrow or spleen or lymph nodes, low or absent NK-cell activity, and an elevated level of soluble cluster of differentiation (CD) 25 [8].

Our patient had a history of warm AIHA for which she was on immunosuppressive therapy (prednisolone and azathioprine). AIHA is an uncommon disorder with an estimated incidence rate of 1-3 cases per 100000 per year and characterized by the production of antibodies directed against self-red blood cells [9,10]. We believed that the immunologic mechanisms involved in the pathogenesis of AIHA could predispose to HLH in this case. An unusual association between HLH, mixed connective tissue disease, and AIHA was reported previously by Kelkar et al. [11]. However, the development of HLH associated with dengue infection and AIHA has not been reported in the past. In this present case, dengue infection triggered an acute hemolytic episode in a patient with warm AIHA, and the course was then complicated by HLH. Despite early recognition of HLH and prompt treatment with corticosteroids and intravenous immunoglobulin, we were unable to reverse the severe, refractory HLH which eventually resulted in fatal outcome.

Dengue infection has various bleeding manifestations, ranging from asymptomatic petechial skin hemorrhage to life-threatening cerebral, pulmonary, and gastrointestinal hemorrhages [12]. Retroperitoneal hematoma is a very rare hemorrhagic complication of dengue infection and has only been described in several case reports [12,13]. 

## Conclusions

HLH is an uncommon but severe complication in dengue infection whereby, in this case, AIHA might be an important predisposing factor. Early recognition and prompt treatment may improve patient outcomes in dengue-associated HLH. Severe dengue can be complicated by bleeding at the atypical site such as retroperitoneal hematoma. Hence, clinicians must be more vigilant of this severe, life-threatening complication in any patients presenting with abdominal distension or pain, rapid drop in hemoglobin, and dengue shock syndrome. Transfusion of blood products, correction of coagulopathy, and timely surgical intervention may improve outcomes.
